# Collagen remodeling in murine melanoma therapy response through second-harmonic generation imaging

**DOI:** 10.1117/1.BIOS.1.3.030501

**Published:** 2024-12-17

**Authors:** Mihaela Balu

**Affiliations:** University of California, Irvine, Beckman Laser Institute and Medical Clinic, Department of Dermatology, Irvine, California, United States

## Abstract

The study by Heaton et al. marks a significant advancement in understanding the role of collagen remodeling within the melanoma tumor microenvironment during immunotherapy. Using in vivo second-harmonic generation imaging, the authors quantitatively tracked dynamic changes in collagen morphology in a preclinical melanoma model, revealing a shift toward a healthier phenotype associated with treatment. These findings enhance our understanding of tumor extracellular matrix dynamics and highlight the potential of optical imaging technologies to guide and optimize cancer immunotherapy. This commentary will explore these findings, contextualize them within the broader field of tumor immunology, and discuss their implications for improving immunotherapy strategies in melanoma and other cancers.

Wouldn’t it be remarkable to have a tool that not only visualizes tumor architecture but also quantifies its dynamic changes in response to treatment? While traditional histopathology remains a cornerstone of cancer diagnostics, non-invasive imaging modalities are increasingly enhancing our ability to study tumor biology in real time. Collagen, a critical component of the tumor microenvironment, has long been recognized as both a barrier to immune cell infiltration and a driver of tumor invasion and metastasis through its reorganization and alignment at the tumor-stromal interface.^1^^–^^6^ Despite melanoma’s high fatality rate and significant fibroblast involvement, studies investigating collagen dynamics during treatment have been limited.

Using second-harmonic generation (SHG) imaging and advanced analysis tools, the study by Heaton et al.^7^ investigates collagen remodeling in a melanoma mouse model treated with a combination of radiotherapy, immunotherapy, and immunocytokines, aiming to determine whether treatment shifts collagen toward a healthier phenotype (Fig. 1).

**Fig. 1:**
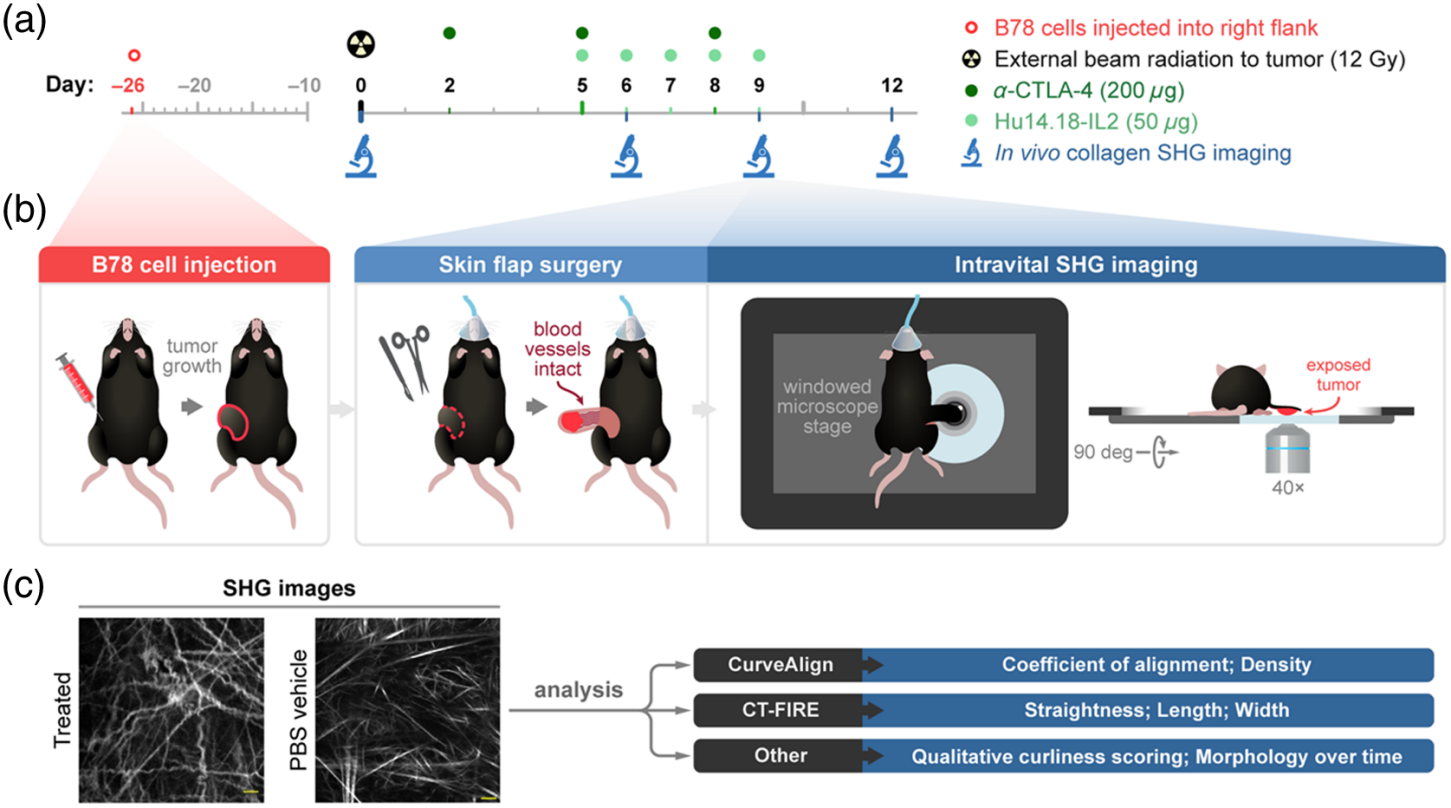
*In vivo* SHG imaging and treatment workflow. (a) Mice were inoculated with B78 melanoma cells and treated with radiation, *α*-CTLA-4, and Hu14.18-IL2, or vehicle control. SHG imaging was performed pre- and post-treatment (days 0, 6, 9, 12). (b) Tumors were surgically exposed for imaging under anesthesia, with each time point using new mice. (c) Collagen images were analyzed with CurveAlign and CT-FIRE to assess morphological changes.

Treated tumors demonstrated significant reorganization of collagen morphology, shifting toward a healthier phenotype characterized by shorter, wider, and curlier fibers compared to untreated controls. Temporal analysis revealed distinct patterns: collagen width and density changes occurred early (day 6), while straightness and length changes appeared later (days 9 and 12), coinciding with tumor regression and immune activation. Quantitative analysis using CurveAlign and CT-FIRE software showed that single-fiber features, such as straightness and width, were more sensitive indicators of treatment response than bulk metrics like alignment and density.

While this study represents significant progress, several challenges and limitations remain to be addressed in future work. SHG imaging alone cannot determine the biological mechanisms driving collagen changes, underscoring the need for complementary techniques such as flow cytometry or RNA sequencing. Furthermore, the specific contributions of each component of the triple therapy to collagen reorganization are unclear, necessitating studies that isolate individual effects. Lastly, as findings from the mouse melanoma model may not fully generalize to other cancers, validation in additional models and clinical settings is essential.

In conclusion, this study demonstrates the potential of SHG imaging for characterizing treatment-induced collagen remodeling and shows promise as a tool for guiding and optimizing melanoma immunotherapy. Beyond its immediate findings, this work opens the door to exploring how changes in the extracellular matrix interact with immune responses during cancer treatment. Recent advances in clinical imaging of melanoma using SHG imaging to visualize collagen and two-photon excited fluorescence to capture cellular structures,^8^^,^^9^ including immune cells,^10^ have shown great potential for translating optical imaging into clinical tools for assessing melanoma and its treatment effects. Integrating such advanced optical imaging modalities with genomics and transcriptomics approaches could pave the way for more precise and effective cancer immunotherapy strategies for melanoma and beyond. Future studies could build on this work to better understand the interplay between collagen remodeling and immune cell activity across different cancers, potentially uncovering new biomarkers or therapeutic targets.
